# German Mobile Apps for Patients With Psoriasis: Systematic Search and Evaluation

**DOI:** 10.2196/34017

**Published:** 2022-05-26

**Authors:** Christian Lull, Jan Alwin von Ahnen, Georg Gross, Victor Olsavszky, Johannes Knitza, Jan Leipe, Astrid Schmieder

**Affiliations:** 1 Department of Dermatology University Medical Center Mannheim Heidelberg University Mannheim Germany; 2 Department of Internal Medicine - Rheumatology and Immunology Friedrich-Alexander University Erlangen-Nürnberg Universitätsklinikum Erlangen Erlangen Germany; 3 Fifth Department of Medicine (Nephrology/Endocrinology/Rheumatology) University Medical Centre Mannheim, Medical Faculty Mannheim University of Heidelberg Mannheim Germany; 4 Department of Dermatology, Venereology, and Allergology University Hospital Würzburg Würzburg Germany

**Keywords:** psoriasis, eHealth, mHealth, telemedicine, teledermatology, disease management, smartphone application, mental health, mobile health, health app, dermatology, skin

## Abstract

**Background:**

Psoriasis is a chronic inflammatory skin disease. The visibility of erythematous plaques on the skin as well as the pain and itchiness caused by the skin lesions frequently leads to psychological distress in patients. Smartphone apps are widespread and easily accessible. Earlier studies have shown that apps can effectively complement current management strategies for patients with psoriasis. However, no analysis of such apps has been published to date.

**Objective:**

The aim of this study is to systematically identify and objectively assess the quality of current publicly available German apps for patients with psoriasis using the Mobile Application Rating Scale (MARS) and compile brief ready-to-use app descriptions.

**Methods:**

We conducted a systematic search and assessment of German apps for patients with psoriasis available in the Google Play Store and Apple App Store. The identified apps were randomly assigned to 1 of 3 reviewers, who independently rated them using the German MARS (MARS-G). The MARS-G includes 15 items from 4 different sections (engagement, functionality, aesthetics, and information) to create an overall mean score for every app. Scores can range from 1 for the lowest-quality apps to 5 for the highest-quality apps. Apps were ranked according to their mean MARS-G rating, and the highest-ranked app was evaluated independently by 2 patients with psoriasis using the user version of the MARS-G (uMARS-G). Furthermore, app information, including origin, main function, and technical aspects, was compiled into a brief overview.

**Results:**

In total, we were able to identify 95 unique apps for psoriasis, of which 15 were available in both app stores. Of these apps, 5 were not specifically intended for patients with psoriasis, 1 was designed for clinical trials only, and 1 was no longer available at the time the evaluation process began. Consequently, the remaining 8 apps were included in the final evaluation. The mean MARS-G scores ranged from 3.51 to 4.18. The app with the highest mean MARS-G score was Psoriasis Helferin (4.18/5.00). When rated by patients, however, the app was rated lower in all subcategories, resulting in a mean uMARS-G score of 3.48. Most apps had a commercial background and a focus on symptom tracking. However, only a fraction of the apps assessed used validated instruments to measure the user’s disease activity.

**Conclusions:**

App quality was heterogeneous, and only a minority of the identified apps were available in both app stores. When evaluated by patients, app ratings were lower than when evaluated by health care professionals. This discrepancy highlights the importance of involving patients when developing and evaluating health-related apps as the factors that make an app appealing to users may differ between these 2 groups.

**Trial Registration:**

Deutsches Register Klinischer Studien DRKS00020963; https://tinyurl.com/ye98an5b

## Introduction

Psoriasis is a chronic inflammatory skin disease affecting about 1.5 million people in Germany [1]. Erythematosquamous plaques, mostly on extensor surfaces of the extremities, are characteristic of this illness, but the disease can involve every part of the skin and can also affect the joints.

The chronicity of psoriasis and the pain, itchiness, and stigma associated with it put an immense physical and mental burden on patients. In addition, psoriasis is associated with several comorbidities, including diabetes [2], cardiovascular disease [3], inflammatory bowel disease [4], anxiety, and depression [5].

Although there is currently no known cure for the disease, a wide variety of treatment options, ranging from phototherapy to topical and systemic agents, are available and highly effective in alleviating signs and symptoms in most patients [6]. However, adherence to treatment as well as knowledge of the disease and its optimal management are often low [7], potentially diminishing treatment efficacy [8].

The rise of smartphone use in the general population in recent years opens new possibilities for the care of patients with dermatological conditions. mHealth provides unprecedented and personalized tools to complement and boost existing therapies [9], as highlighted in a study by Svendson et al [10]. The authors demonstrated that smartphone apps targeted specifically at patients with psoriasis led to a significant improvement in adherence to treatment and outcomes [10]. Another study on patients with rheumatic diseases by Knitza et al [11] showed that most participants saw medical apps as beneficial and would use such apps if available. It is plausible to assume that such a survey among patients with psoriasis would yield similar results given that both conditions are chronic, difficult to treat, and associated with low adherence to existing treatments [7]. In 2019, Germany established the digital health applications (DiGA) directory, where scientifically validated digital health apps licensed as medical devices are listed systematically. Similar to medications, physicians can now prescribe DiGAs, and costs are reimbursed by insurance companies. However, at the moment, no DiGAs exist for patients with psoriasis. Therefore, these patients and their treating dermatologists are still confronted with a confusingly large number of apps, offering different functions and modalities; these apps are often not evidence-based and ineligible for cost reimbursement by insurance companies [12,13].

The goal of this study was therefore to identify and assess publicly available smartphone apps for patients with psoriasis and create brief app descriptions, including objective quality ratings. To our knowledge, a systematic review and assessment of smartphone apps for patients with psoriasis has not been conducted to date.

## Methods

### App Screening

We conducted a systematic search of the German Apple App Store as well as the Google Play Store on January 7, 2021. The search terms used were as follows: “Psoriasis” OR “Schuppenflechte” (the German nonmedical term for psoriasis). A total of 2 independent reviewers searched each app store. The inclusion criteria are as follows: apps that were (1) available in both app stores, (2) available in the German or English language, and (3) specifically designed for patients with psoriasis. The exclusion criteria are as follows: apps that (1) were designed for conferences or clinical trials, (2) were not free to use, and (3) included advertisements.

### App Characteristics

We collected the following information available in the app stores and on app homepages:

- App name
- Rating
- Number of ratings
- Developer
- Version
- Date of last update
- Cost
- Platform and affiliations

We collected the following information on the apps from the app stores or the developer’s website:

- Affiliation (commercial, government, nongovernmental organization [NGO], university, or not known)
- Focus (increase happiness or well-being; mindfulness, meditation, or relaxation; reduce negative emotions; depression; anxiety or stress; anger; behavior change; alcohol or substance use; goal setting; entertainment; relationships; physical health; or other)
- Theoretical background (assessment; feedback; information or education; monitoring or tracking; goal setting; advice, tips, strategies, or skills training; cognitive behavioral therapy (positive events and thought challenging); acceptance commitment therapy; mindfulness or meditation; relaxation; gratitude; strengths-based; or other)
- Technical aspects (allows sharing, has an app community, allows password protection, requires login, sends reminders, and needs web access to function)

### App Quality Ratings

The 3 reviewers used the validated German version of the Mobile Application Rating Scale (MARS-G) [14,15], and all reviewers previously underwent training on how to correctly apply the MARS to app evaluation using a training video [16], as suggested by Stoyanov et al [17].

The MARS score captures the following 4 objective aspects:

- Engagement (5 items)
- Functionality (4 items)
- Aesthetics (3 items)
- Information (7 items)

These sections contain a total of 19 items on a 5-point Likert scale, from 1 (strongly disagree) to 5 (completely agree), as well as a subjective measure of app quality with 4 additional questions. The final MARS-G score for each app is calculated as the mean of the 4 objective categories (engagement, functionality, aesthetics, and information). The score can range between 1 (worst) to 5 (best). The subjective app quality is additionally reported as the mean score of the 4 respective questions.

In addition, the MARS-G includes an app-specific subjective perceived impact score, called the psychotherapy score. The psychotherapy score includes the following 6 items: awareness, knowledge, attitudes, intention to change, help-seeking, and behavior change. These items can be used to estimate the app’s impact on knowledge, attitudes, and intention to change behavior.

For training purposes, the MARS-G was used by all 3 reviewers to evaluate 1 app that was excluded from the study based on our inclusion and exclusion criteria. The results were discussed until no questions remained to achieve the same understanding across reviewers.

We randomly assigned each app to 2 reviewers. Of the reviewers, 2 used an iPhone (iPhone X and iPhone 12 Pro, both running with iOS 14.3; Apple Inc) and 1 used an Android phone (Asus ZenFone 3 running Android 8.0.0; ASUSTek Computer Inc). We rated all apps included in this study from January 9, 2021, to January 29, 2021. As required, every app was tested independently for at least 10 minutes before applying the MARS-G criteria.

All reviewers were medical students aged 22 to 25 years who were focusing on patients with psoriasis as part of their medical studies.

Additionally, the best-rated app was evaluated by 2 patients with psoriasis at the Department of Dermatology, Venereology, and Allergology at the University Medical Center Mannheim with the user version of the MARS-G (uMARS-G), a modified version, specifically for patients [18]. There are only a few differences between the uMARS-G and MARS-G. The information category contains 4 questions instead of 7, and the uMARS-G completely omits the psychotherapy score.

Patients were asked to spend at least 10 minutes exploring the app before rating it.

### Ethics Approval

The study was conducted in accordance with the Declaration of Helsinki and approved by the Medical Ethics Committee of the Medical Faculty Mannheim, Heidelberg University (2020-515N-MA). The trial is registered at Deutsches Register Klinische Studien (registration number DRKS00020963). Written informed consent was provided by each patient before participating in the study.

### Patient Characteristics

The selected patient participants were already part of other trials in which they also used a medical health app. Both seemed to be reliable and conscientious when answering questionnaires. Likewise, both could be assumed to have sufficient language comprehension and competence. One of the patients was 38 years of age and the other was 56 years. Both were asked to participate in the survey on March 8, 2021, during their appointments at the dermatology outpatient clinic.

### Statistical Analysis

After assessing the MARS-G score for all apps using the previously described methods, the results from both raters were averaged to represent the final score each app achieved in our study.

## Results

### App Screening

**Figure 1:**
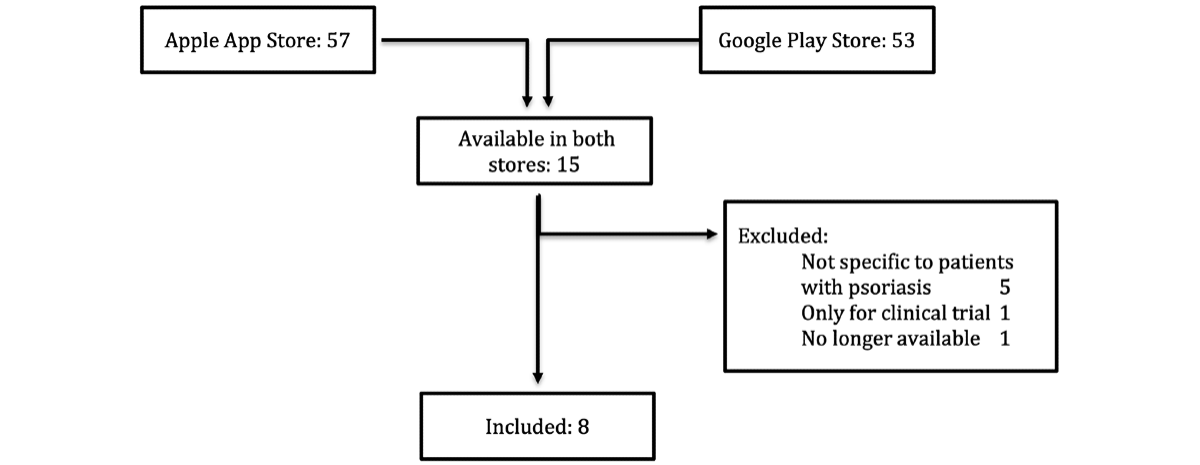
App screening process.

A total of 95 unique apps were identified in the German Apple App Store (n=57) and the Google Play Store (n=53) using the previously specified search terms. 15 of the apps were available in both stores. Of these apps, 5 were not specifically targeted at patients with psoriasis and 1 was designed for a clinical trial only. Furthermore, 1 app that was previously included in our study was no longer available in both app stores at the time of rating. Therefore, the app was excluded from further analyses. A total of 9 apps were eligible for our study (Figure 1).

### App Characteristics

A majority of the apps (6/8, 75%) were commercial, password-protected (6/8, 75%), and focused on symptom tracking (ie, diaries, 5/8, 63%; Table 1). The remaining 2 apps were affiliated with an NGO (1/8, 13%) or of unknown origin (1/8, 13%). Of the 8 apps, 2 (25%), Itchy – Psoriasis & Ekzem and DLQI 4 Psoriasis, used scientifically validated scores and questionnaires to evaluate the patient’s condition, such as the Psoriasis Area and Severity Index (PASI) [19] and Dermatology Life Quality Index (DLQI) [20]. The remaining apps, which offered diary functions, did not use validated instruments.

Of the 8 apps, 3 (38%) allowed users to connect with other patients with psoriasis through an app community. The Kopa for Psoriasis app additionally offers disease information and recommendations; however, the sources of the information were not indicated. The P.S.O. Psoriasis Arztfinder acts as a search engine, enabling users to find German physicians treating patients with psoriasis.

**Table 1:** Origin, focus, and specific technical aspects of the apps included in the evaluation.

App name	Origin	Focus	Theoretical background	Technical aspects
DLQI 4 Psoriasis	Commercial	Symptom diary	DLQI^a^	N/A^b^
Imagine – Skin Tracker	Commercial	Symptom diary	N/A	N/A
Itchy – Psoriasis & Ekzem	Unknown	Symptom diary	DLQI, PASI^c^	Allows password-protection
Kopa for Psoriasis	Commercial	Web-based forum, information	N/A	Has an app community, allows password-protection
P.S.O. Psoriasis Arztfinder	NGO^d^	Finding physicians	N/A	Allows password-protection
Psoriasis Forum	Commercial	Web-based forum	N/A	Has an app community, allows password-protection
Psoriasis Helferin	Commercial	Symptom diary	N/A	Allows password-protection
Psoriasis Monitor	Commercial	Symptom diary	N/A	Allows sharing on social media, has an app community, allows password-protection

^a^DLQI: Dermatology Life Quality Index.

^b^N/A: not applicable. These apps did not use validated instruments and therefore have no theoretical background.

^c^PASI: Psoriasis Area and Severity Index.

^d^NGO: nongovernmental organization.

### App Quality Ratings

Table 2 shows the apps’ MARS-G ratings. The mean MARS-G score for all assessed apps varied between 3.00 and 4.18. The app Psoriasis Helferin received the highest MARS-G score (4.18), followed by Imagine – Skin Tracker (4.08) and Psoriasis Forum (4.01). The highest psychotherapy subscale score was achieved by Psoriasis Helferin (3.50) and Imagine – Skin Tracker (3.50), followed by Psoriasis Monitor (3.25). The highest MARS-G subjective scores were achieved by Imagine-Skin Tracker (3.88) and Psoriasis Helferin (3.25). The interrater reliability was 0.66.

**Figure 2:**
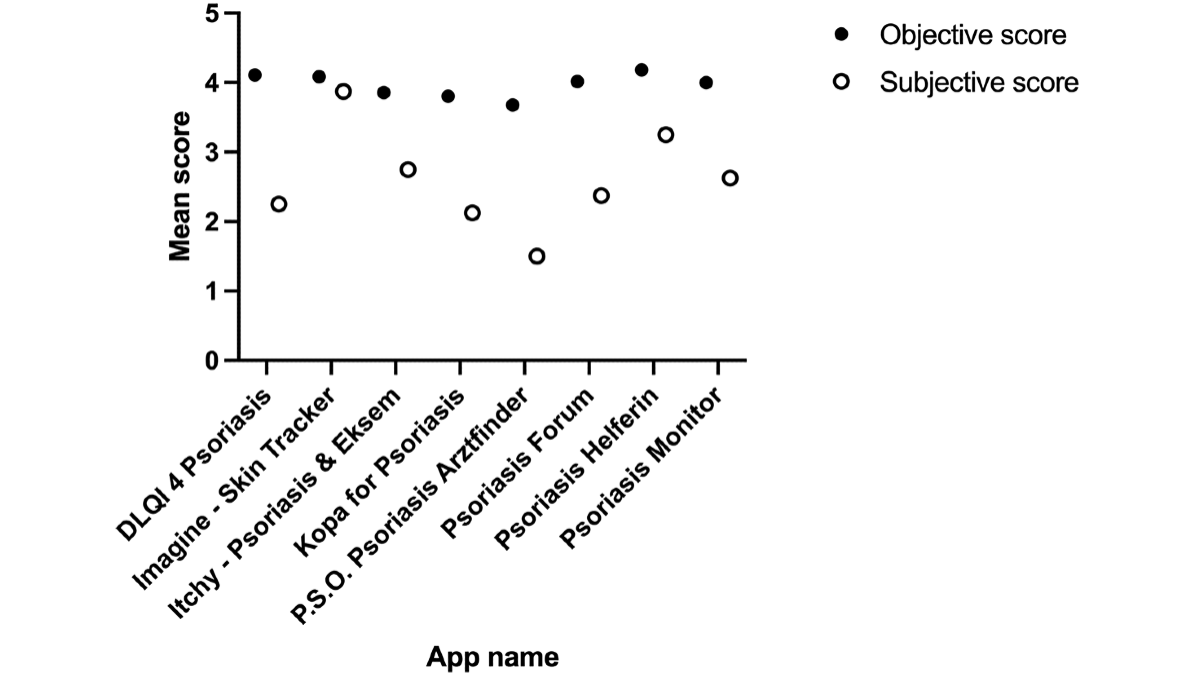
Mean objective and subjective German Mobile Application Rating Scale (MARS-G) scores.

**Figure 3:**
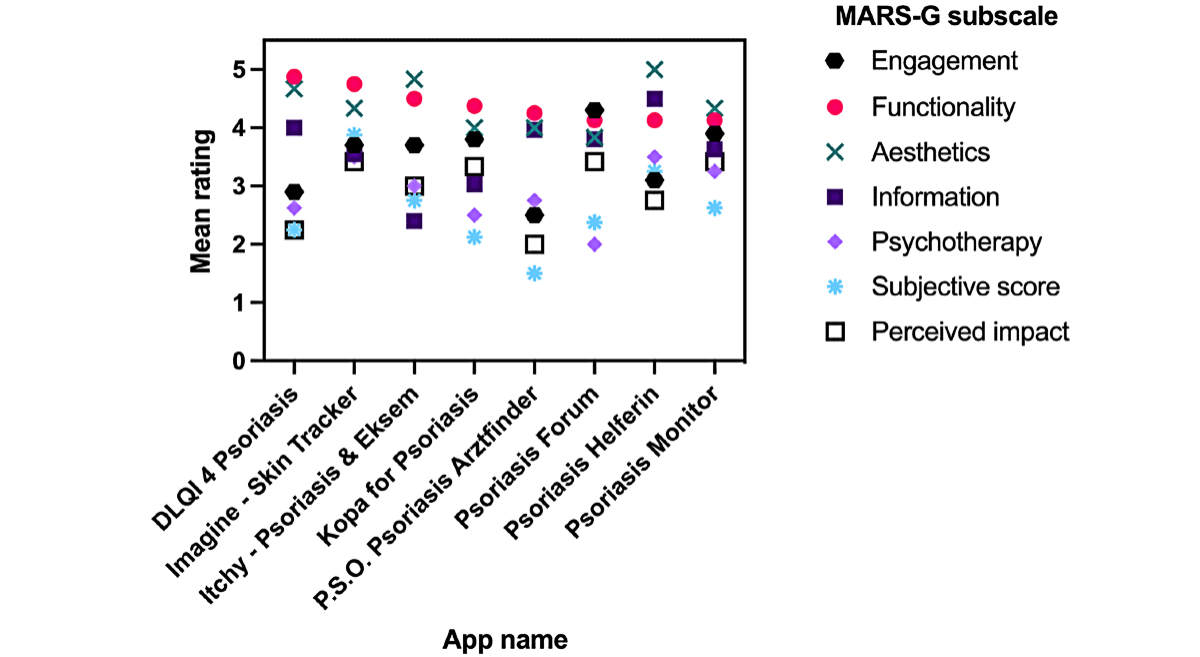
Mean scores for each German Mobile Application Rating Scale (MARS-G) subscale.

When comparing the objective MARS-G score and the subjective subscale scores, all apps received a higher objective MARS-G rating (Figure 2). A detailed analysis of the mean subscale ratings across all apps revealed that the apps were rated best in aesthetics and functionality (4.31; Figure 3). In contrast, the apps achieved the lowest ratings for psychotherapy (2.792) and the subjective score (2.21).

**Table 2:** App version, general function, and mean app quality calculated by professional raters using the German Mobile Application Scale (MARS-G).

App name	Version			General function		Engagement^a^		Functionality^a^		Aesthetic^a^		Information^a^		Mean objective score^a^		Mean subjective score^a^		Psychotherapy^a^
	iOS	Android																
DLQI 4 Psoriasis	1.0	1.0	Progress documentation		2.90		4.88		4.67		4.00		4.11		2.25		2.63	
Imagine – Skin Tracker	2	N/A^b^	Progress documentation		3.70		4.75		4.33		3.55		4.08		3.88		3.50	
Itchy – Psoriasis & Ekzem	1	N/A	Progress documentation		3.70		4.50		4.83		2.40		3.86		2.75		3.00	
Kopa for Psoriasis	4.80	N/A	Forum		3.80		4.38		4.00		3.03		3.80		2.13		2.50	
P.S.O. Psoriasis Arztfinder	0	N/A	Finding physicians		2.50		4.25		4.00		3.97		3.68		1.50		2.75	
Psoriasis Forum	0	1.0.3	Forum		4.30		4.13		3.83		3.80		4.01		2.38		2.00	
Psoriasis Helferin	4.7	1.0.1	Progress documentation		3.10		4.13		5.00		4.50		4.18		3.25		3.50	
Psoriasis Monitor	4.4	2.0.12	Progress documentation, communication with a physician		3.90		4.13		4.33		3.63		3.00		2.63		3.25	

^a^Each score is based on the MARS-G.

^b^N/A: not applicable. Both raters used an iOS phone to rate these apps.

### uMARS-G Ratings

**Figure 4:**
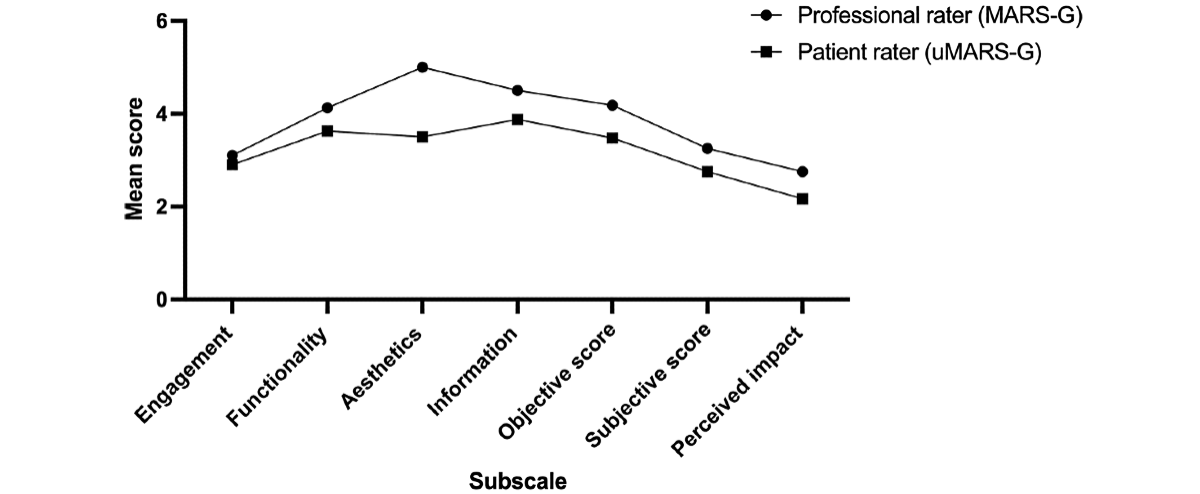
Mean subscale scores for the German Mobile Application Rating Scale (MARS-G) and user version of the MARS-G (uMARS-G).

As Psoriasis Helferin received the highest MARS-G score (4.18), it was then rated by 2 patients with psoriasis, resulting in a considerably lower uMARS-G score (3.48). Patient ratings were lower for all uMARS-G subscales (Figure 4).

## Discussion

### Principal Findings

To our knowledge this is the first study systematically identifying and rating currently available German smartphone apps specifically designed for patients with psoriasis. App quality was assessed by independent reviewers and patients using validated instruments and ready-to-use information was compiled to inform patients and health care professionals.

The overall app quality was heterogeneous. The app Psoriasis Helferin achieved the highest MARS-G score (4.18), and its main function is to track symptoms.

When 2 patients with psoriasis rated this app using the uMARS-G, the mean score decreased to 3.48. All subcategories were scored lower by patients (uMARS-G) than by professionals (MARS-G). The aesthetics subcategory revealed the largest difference.

These rating differences demonstrate the different perceptions, priorities, and preferences of patients. Therefore, health care providers should offer their patients a selection of apps or, at least, customizable apps. Patients may use medical apps less often if their preferences are not considered; this has already been demonstrated in studies analyzing treatment adherence [21]. This topic must be explored further in clinical studies.

Psoriasis Helferin also achieved the best results in the MARS-G information subscale, with 4.50 points. Importantly, Psoriasis Helferin does not include any validated disease assessment instruments such as the PASI or DLQI [19,20]. This makes its clinical use problematic since there is no established procedure to date for comparing data collected by an app to medical records produced during routine visits. In our opinion, more apps that include scientifically validated instruments are required to increase the validity of patient-generated data.

We showed that all apps achieved passable results in the dimensions of aesthetics and functionality. By contrast, only 2 apps achieved 4 points or more for the information dimension, DLQI 4 Psoriasis and Psoriasis Helferin; no apps achieved 4 points or more for the psychotherapy dimension. This could indicate that app developers do not focus sufficiently on providing evidence-based information and psychological support to patients. It has been shown that emotional well-being is higher in well-informed patients [22]. In addition, the willingness to seek help from qualified physicians and change one’s behavior are important precursors to successful treatment. Therefore, the questions addressed in the psychotherapy score determine if the app will be able to help patients improve their conditions. For the psychotherapy dimension, Psoriasis Helferin also achieves a passable score, along with the app Imagine – Skin Tracker, achieving 3.50 points.

Similar to previous app reviews [22], our results highlight the importance of including patients, clinicians, and researchers in the app development process, as stressed previously, to create appealing, validated, and truly beneficial apps. Physicians should be aware of the content and quality of the apps they recommend or even prescribe. In this regard, apps that primarily include a forum function should be approached with caution since personal experiences and incorrect advice from unqualified users may be unfavorable to the medical management of the patient’s condition. Interestingly, an earlier study among patients with rheumatic conditions showed that this group was the least interested in a forum function [11]. Although they also live with a chronic disease, it remains to be seen if this conclusion can be transferred to patients with psoriasis. Thus, further surveys on the preferences of patients with psoriasis are needed to identify the most important app subjects and functions.

### Limitations

The MARS is one of the most often used and validated tools to evaluate health app quality [14]. The interrater reliability was 0.66 in our study, showing moderate agreement between raters.

The MARS helps raters evaluate the functionality, aesthetics, and information provided by apps; however, we found the equal contribution of all 4 categories to the final score is suboptimal for certain types of apps. For example, for an app focusing on information for patients, the quality of the information provided should have more weight than for an app used solely as a symptom diary. In apps designed with a narrow focus, the final score does not necessarily reflect the overall quality of these apps. Thus, we recommend using the MARS only to compare apps with a similar focus.

Further, data privacy and security are not part of the MARS, although it is an important aspect in any analysis of health care apps with sensitive information being shared by users. Although all apps but Kopa for Psoriasis included a privacy statement that the user had to agree to before use, the statements were long and difficult to understand for the average user. This makes it challenging for any patient or health care provider to grasp where and how their data is stored.

In an article from 2017, Baptista et al [23] question the utility of the uMARS as a simple adaptation of the MARS for lay users since the perceived quality of mobile apps may differ widely between health care providers and patients. This difference in perception can also be seen in our data, where the aesthetics of the apps were rated much lower by patients compared to researchers. We agree with Baptista et al [23] that further research addressing the different perspectives of patients and health care providers is needed.

Another limitation is that by focusing on apps available in app stores, we excluded web-based apps. The decision to only include apps only available in both app stores was based on our aim to analyze apps which are easily accessible and may be recommended by physicians. However, this approach excluded a significant proportion of apps available in only 1 of the app stores and makes our results less generalizable. In addition, the digital world is constantly changing; therefore, the results of this study may only be relevant for a short period of time, necessitating the frequent reanalysis of the key data.

### Conclusions

We were able to identify and compile several German apps specifically designed for patients with psoriasis that are publicly available and free of charge. Using the MARS-G, the highest mean score was achieved by Psoriasis Helferin. Importantly, patients rated the apps less positively than health care professionals. This should be considered when digital health care apps for patients with psoriasis become available on prescription as part of the DiGA directory in Germany. To be considered as DiGA, however, studies on the efficacy of specific apps are needed, which so far do not exist for all the apps we evaluated. Both professionals and patients rated the perceived impact of Psoriasis Helferin on health behavior as moderate. Other apps, which were evaluated by professionals only, performed even better in this area. Thus, we conclude that the benefit of apps as complements to traditional therapy for patients with psoriasis can not only be determined by randomized controlled trials [10] but also through subjective evaluations by patients and professionals. Additionally, a greater emphasis should be put on the evaluation of data privacy as private and often sensitive data are shared through these apps. Mobile dermatology apps represent a promising tool to complement the care of patients with psoriasis, but many critical aspects must be analyzed in more detail in an interdisciplinary manner, requiring close collaboration between dermatologists, app developers, and data protection officers.

## Appendix

Multimedia Appendix 1 CONSORT-eHEALTH checklist (V 1.6.1).

## Abbreviations

DiGA: digital health applications
DLQI: Dermatology Life Quality Index
MARS: Mobile Application Rating Scale
MARS-G: German Mobile Application Rating Scale
NGO: nongovernmental organization
PASI: Psoriatic Area and Severity Index
uMARS: user version of the Mobile Application Rating Scale
